# Striatal insights: a cellular and molecular perspective on repetitive behaviors in pathology

**DOI:** 10.3389/fncel.2024.1386715

**Published:** 2024-03-27

**Authors:** Charlotte Lauren Burton, Alessandra Longaretti, Andjela Zlatanovic, Guilherme Monteiro Gomes, Raffaella Tonini

**Affiliations:** Neuromodulation of Cortical and Subcortical Circuits Laboratory, Istituto Italiano di Tecnologia, Genoa, Italy

**Keywords:** striatum, epigenetics, repetitive behavior, cell-type specificity, obsessive-compulsive disorder, autism spectrum disorder, Gilles de la Tourette syndrome, Huntington’s disease

## Abstract

Animals often behave repetitively and predictably. These repetitive behaviors can have a component that is learned and ingrained as habits, which can be evolutionarily advantageous as they reduce cognitive load and the expenditure of attentional resources. Repetitive behaviors can also be conscious and deliberate, and may occur in the absence of habit formation, typically when they are a feature of normal development in children, or neuropsychiatric disorders. They can be considered pathological when they interfere with social relationships and daily activities. For instance, people affected by obsessive-compulsive disorder, autism spectrum disorder, Huntington’s disease and Gilles de la Tourette syndrome can display a wide range of symptoms like compulsive, stereotyped and ritualistic behaviors. The striatum nucleus of the basal ganglia is proposed to act as a master regulator of these repetitive behaviors through its circuit connections with sensorimotor, associative, and limbic areas of the cortex. However, the precise mechanisms within the striatum, detailing its compartmental organization, cellular specificity, and the intricacies of its downstream connections, remain an area of active research. In this review, we summarize evidence across multiple scales, including circuit-level, cellular, and molecular dimensions, to elucidate the striatal mechanisms underpinning repetitive behaviors and offer perspectives on the implicated disorders. We consider the close relationship between behavioral output and transcriptional changes, and thereby structural and circuit alterations, including those occurring through epigenetic processes.

## 1 Introduction

Repetitive behavior is an umbrella term that includes subsets of normal or pathological repetitive behaviors, tics, and habitual behaviors. While repetitive behaviors are typical in normative development (Thelen, 1979; Evans et al., 1997; Whitehouse and Lewis, 2015), actions performed frequently that do not serve a function or are contextually inappropriate, inflexible, compulsive, or stereotyped, and often negatively affect a person’s life, are considered pathological (Langen et al., 2011). This can be the case in obsessive-compulsive disorder (OCD), autism spectrum disorder (ASD), Huntington’s disease (HD) and Gilles de la Tourette syndrome (GTS; Worbe et al., 2010; Gillan et al., 2011, 2014; Alvares et al., 2016; Hartmann and Millet, 2018; Oosterloo et al., 2019; Zadegan et al., 2024) On the other hand, habitual behaviors are automated responses to a reward or stimulus that are developed by continuous repetitions. They are inflexible and are unaffected without the presence of the reward or when its value is changed (Balleine and O’Doherty, 2010). To describe symptoms of the aforementioned pathologies, terms like ‘repetitive behaviors,’ ‘restricted and repetitive behaviors’ (RRB), and ‘stereotypies’ often take precedence. We concede that a considerable amount of the neural evidence substantiating habitual control of behavior is relevant for understanding the evolution of pathological repetitive behaviors, especially when habit formation is maladaptive. The use of pharmacological and genetic animal models recapitulating key phenotypes of these neuropsychiatric disorders have been instrumental in informing the neural basis of repetitive behavior. In this review, we focus on the neural underpinnings of pathological repetitive behaviors inferred from human and animal work, while recognizing that this field has substantial overlap with habitual behavior research.

**Figure 1:**
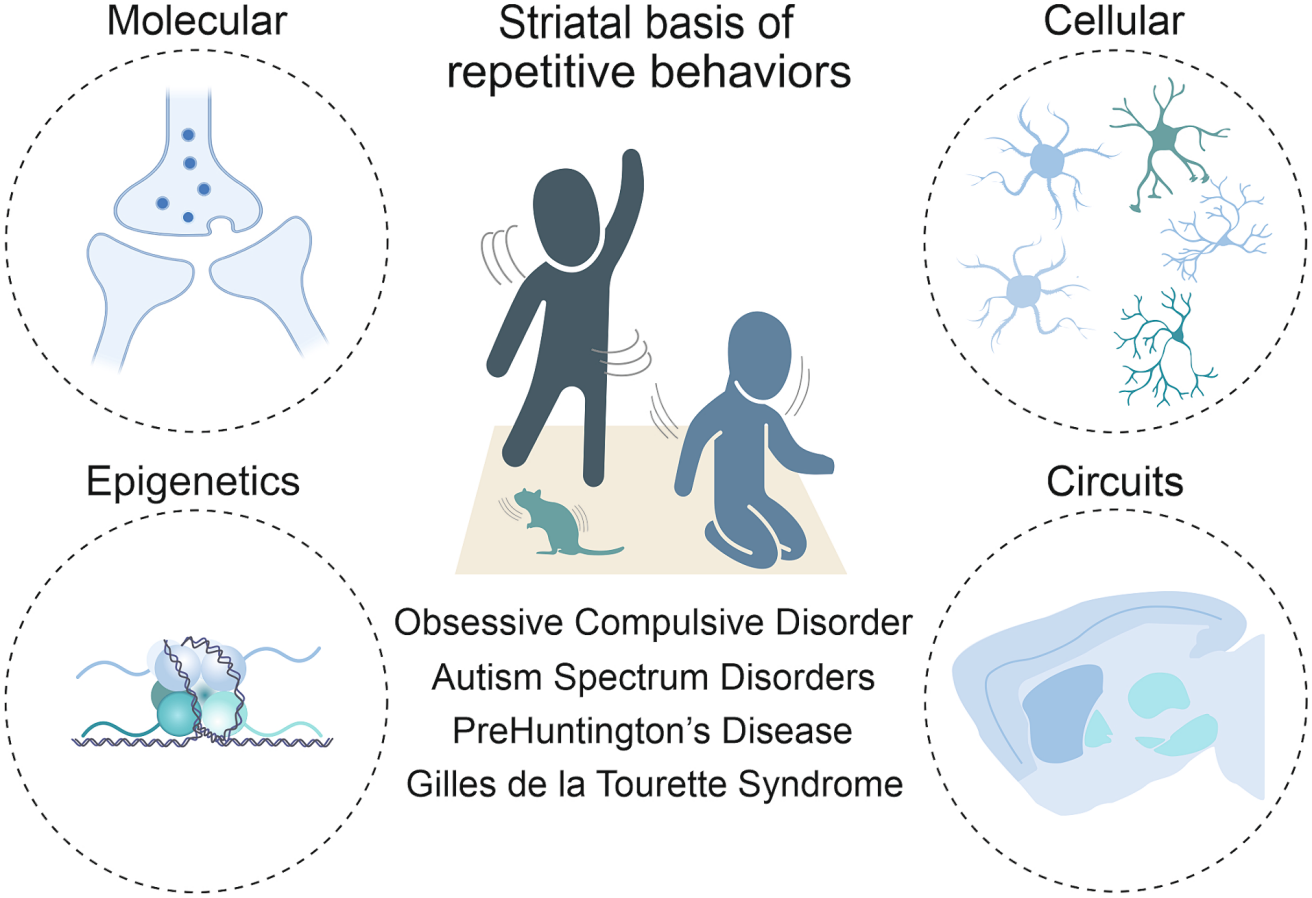
Toward a multi-level understanding of striatal dysfunctions underlying repetitive behaviors in neurodevelopmental, neuropsychiatric and neurodegenerative diseases (i.e., Obsessive-Compulsive Disorder, Autism Spectrum Disorder, premanifest Huntington’s Disease and Gilles de la Tourette Syndrome).

Anatomically placed at the hub of the basal ganglia network of the rodent and human brain, the striatum, with its input and output areas, represents a key brain region for the initiation and control of movements, as well as for the selection and reinforcement of actions that can become repetitive or habitual. Indeed, impairments in striatal functions have been associated with a variety of repetitive behavioral phenotypes across different pathologies. In light of this, it is critical to move beyond a strictly neuroanatomical or neuromodulatory perspective of striatal influence (Lewis et al., 2007) and consider the wider neurobiological context to fully understand these behaviors. As such, this review includes the corticostriatal circuit level details first, then the contributions of the main cells in the striatum, being direct and indirect spiny projection neurons (dSPNs and iSPNs) and their molecular underpinnings. It delves into the role of macro striatal organization in striosomes and matrix compartments, and the influence of interneurons and astrocytes. Finally, given the development of repetitive behaviors in these pathologies is not well understood, but it is known that the environment plays a role in their manifestation, we sought to include epigenetic findings. Indeed, chromatin and RNA modifications, as well as non-coding RNA species, may represent a bridge between environmental stimuli and changes at the transcriptional level. These changes can ultimately modify cellular processes, and therefore circuit physiology (Figure 1).

## 2 Corticostriatal and subcortical network in the regulation of repetitive behaviors

Acting requires the convergence of motivational, sensorimotor, and cognitive signals in the striatal hub of the basal ganglia. The striatum receives glutamatergic inputs from the cortex, thalamus, and limbic regions of the brain, as well as dopaminergic afferents from the subcortical regions of the substantia nigra pars compacta (SNc) and ventral tegmental area (VTA), and serotonergic projections from the dorsal raphe nucleus (DRN). The dorsal striatum is anatomically and functionally dichotomous, consisting of the dorsomedial striatum (DMS), also referred to as the cognitive or associative striatum, and the dorsolateral striatum (DLS), known for its role in sensorimotor processing (Yin et al., 2004; Balleine et al., 2007, 2009; Balleine and O’Doherty, 2010; Thorn et al., 2010). Conversely, the ventral striatum (VS) is often characterized as the motivational striatum, anchoring the reward-related aspects of behavior (Berke, 2018). In humans, the dorsal striatum is composed by the caudate and putamen, while the ventral striatum includes the nucleus accumbens (NAc) and the ventromedial portions of the caudate and putamen. As in the rodent brain, input regions of the human striatum comprise somatosensory and motor cortices, projecting to the putamen. The prefrontal cortex (PFC) is directly connected to the caudate, and the VTA innervates the NAc. The cortex establishes organized loops that extend from ventromedial to dorsolateral regions, directing information flow from motivational-limbic (specifically lateral orbitofrontal and anterior cingulate) to cognitive (dorsolateral prefrontal) to sensorimotor (motor and oculomotor) pathways within the striatum (Haber et al., 2000; Groenewegen, 2003). Dysregulation in each corticostriatal loop may correlate with a different aspect of repetitive behavior. For example, stereotypies could arise from primarily motor-loop defects, obsessive-compulsive and impulsive behaviors from limbic-loop defects, and persistent, misplaced goal-directed behavior from cognitive-loop defects (for a review, see Langen et al., 2011). Many studies have characterized functional connectivity alterations between regions of the cortex and the striatum for the four pathologies (Ting and Feng, 2011; Kim et al., 2016; Blumenstock and Dudanova, 2020; Lamanna et al., 2023), but what is occurring in the downstream nuclei of the basal ganglia is less well described.

The DMS and the DLS both contain the two types of spiny projection neurons (SPNs; Lu et al., 2021). SPNs of the direct pathway, known as dSPNs, predominantly express the excitatory Gs/olf-coupled D1 dopamine receptors and send projections to the substantia nigra pars reticulata (SNr) and the internal segment of the globus pallidus (GPi). In contrast, SPNs that constitute the indirect pathway, referred to as iSPNs, mainly express the inhibitory Gi/o-coupled D2 dopamine receptors and innervate the external segment of the globus pallidus (GPe), as well as the subthalamic nucleus (STN) and the GPi/SNr. The final stop in both circuits is the thalamus which relays excitatory or inhibitory signals back to the frontal cortex for motor, limbic and executive functions. It is suggested that striatal regions may integrate dopaminergic inputs via the SNc in a similar way as the cortex, in a spiraling circuit from the core of the nucleus accumbens in the ventral striatum to the DMS and then to the DLS (Haber et al., 2000; Yin and Knowlton, 2006; Lerner, 2020; Lüscher et al., 2020). There is still debate about how information flows between these regions, and particularly whether an imbalance between them necessitates the emergence of repetitive behavior. However, it is well known that the striatum is the cornerstone for both physiological and pathological manifestations of repetitive behaviors.

## 3 Striatal circuit alterations in repetitive and habitual behaviors across neurodevelopmental, psychiatric and neurodegenerative disorders

**Figure 2:**
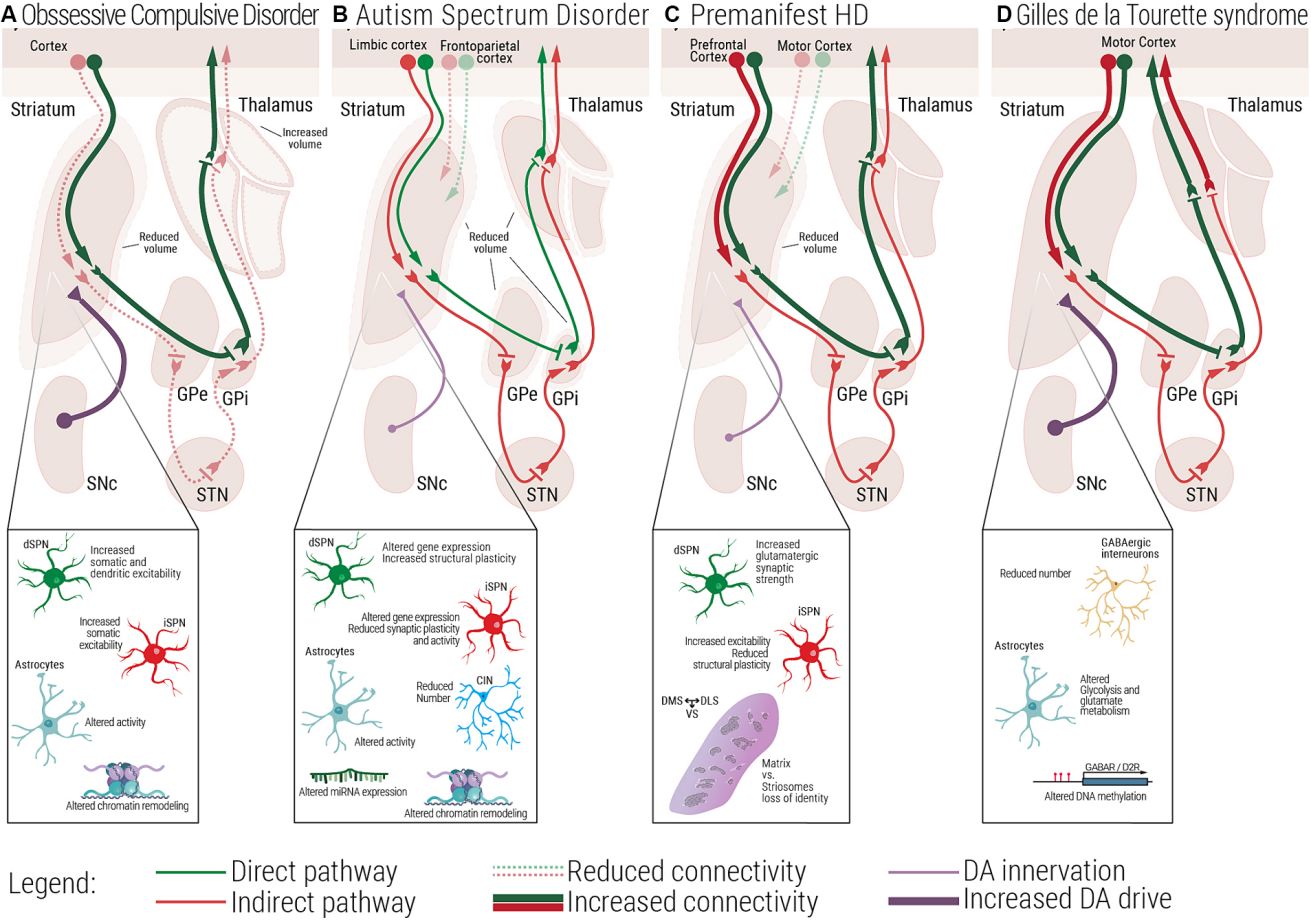
Multiscale evidence for striatal circuitry alterations underlying repetitive behaviors. Diseases like **(A)** Obsessive-Compulsive Disorder, OCD, **(B)** Autism Spectrum Disorder, ASD, **(C)** premanifest Huntington’s Disease, (preHD) and **(D)** Gilles de la Tourette Syndrome, GTS, despite having different etiologies, all share repetitive behaviors as a core symptom in affected patients. Changes at the molecular and cellular level can lead to dysfunctional structural and functional connectivity across the striatal circuitry. **(A)** In OCD, alterations in the electrophysiological properties of dSPNs, iSPNs and astrocytes, can lead to a direct vs. indirect pathway imbalance. **(B)** In ASD, complex alterations at the molecular and cellular level, as well as structural and functional connectivity have been associated with the emergence of repetitive behaviors. **(C)** In premanifest HD, loss of matrix and striosomes cellular identity, as well as altered connectivity between cortical and striatal regions have been reported, at very early stages of the disease. **(D)** In GTS alterations at the cellular and circuit level lead to a scenario where disinhibition of the direct pathway could be the underlying cause of uncontrollable tics. dSPN, direct spiny projection neuron; iSPN, indirect spiny projection neuron; CIN, Cholinergic interneuron; FSI, Fast spiking interneuron; SNc, Substantia nigra pars compacta; GPe, External globus pallidus; GPi, Internal globus pallidus; STN, Subthalamic nucleus; DMS, Dorsomedial striatum; DLS, Dorsolateral striatum; VS, Ventral striatum.

Some of the literature on cellular or circuit-level mechanisms of repetitive behaviors in a pathological state (i.e., ASD, OCD, HD, and GTS; Figures 2A–D) have described habitual components to these behaviors, or have investigated paradigms of habitual behavior. In these contexts, habitual behaviors are considered maladaptive if they persist in the face of punishment, or when a flexible goal-directed behavior (GDB) is more appropriate. This inflexibility aspect is often studied as the pathological behavior in addiction and OCD research (Gillan et al., 2011, 2014). When repetitive behaviors in pathology carry a habitual component, it could be based on their imbalance with GDB in the striatum (Table 1).

**Table 1:** The table summarizes striatal-based alterations across pathologies and species.

Level	Organism	Alteration	Reference
Anatomical alterations			
OCD	Mouse model Slitrk5, Human	Structural changes	Shmelkov et al. (2010) and Piras et al. (2015)
ASD and FXS	Human Mouse model ITSN1/2 ^−/−^	Connectivity changes Structural changes	Shen et al. (2022), Abbott et al. (2018), and Vollweiter et al. (2023)
OCS in preHD	Human	Structural changes	Anderson et al. (2010) and Tabrizi et al. (2011)
	Human	Connectivity changes	Glikmann-Johnston et al. (2021) and McColgan et al. (2015)
GTS	Human	Structural connectivity changes	Church et al. (2009), Worbe et al. (2012, 2015), and Baym et al. (2008)
Striosome and matrix			
ASD and FXS	Mouse model VPA	Aberrant development	Kuo and Liu (2017)
	Mouse model Shank3 ^Δ11/ Δ11^	Signaling imbalance	Ferhat et al. (2023)
	Human	Altered compartmentalization	Kuo and Liu (2020)
OCS in preHD	Mouse model zQ175 Human	Loss of matrix and striosome cell’s identity	Matsushima et al. (2023)
dSPNs and iSPNs			
OCD	Human	Molecular dysfunction	Piantadosi et al. (2021)
	Mouse model Sapap3 ^−/−^	Electrophysiological alterations	Malgady et al. (2023)
	Mouse model Slc1a1^−/−^	Spine density, electrophysiological alterations	Petroccione et al. (2023)
ASD and FXS	Mouse model Shank3	Spine density changes, electrophysiological alterations	Wang et al. (2017)
	Mouse model Shank3 ^Δ11/ Δ11^	Molecular alterations	Ferhat et al. (2023)
	Mouse model VPA	Electrophysiological alterations	Di et al. (2022)
	Mouse model ITSN1/2 ^−/−^	Spine density changes, electrophysiological alterations	Vollweiter et al. (2023)
	Mouse model Fmr1^−/−^	Molecular alterations Spine density changes, electrophysiological alterations	Longo et al. (2023)
OCS in preHD	Human	Cell loss	Reiner et al. (1988) and Deng et al. (2004)
	Mouse model Q175, Htt cKO, YAC128, R6/2, zQ175-Kl	Electrophysiological alterations	Sebastianutto et al. (2017), Burrus et al. (2020), Callahan et al. (2022), and Chambon et al. (2023)
GTS	Human	Transcriptional alterations	Lennington et al. (2016)
Interneurons			
OCD	Mouse model Sapap3^−/−^	PV+	Burguière et al. (2013)
	Mouse model SCIN ablation	CIN	Martos et al. (2017)
ASD and FXS	Mouse model Cntnap2 ^−/−^	PV+	Peñagarikano et al. (2011) and Lauber et al. (2018)
	Mouse model Cntnap2, Shank3, VPA	PV+	Briones et al. (2022)
	Mouse model Cul3 ablation	CIN, PV+	Rapanelli et al. (2017)
	Human	PV+	Dufour et al. (2023)
OCS in preHD	Mouse model R6/2, Lewis rat	CIN	Picconi et al. (2006)
GTS	Human		
	Mouse model SCIN ablation	CIN	Martos et al. (2017)
	Mouse model Cul3 ablation	CIN, PV+	Rapanelli et al. (2023)
Astrocytes			
OCD	Mouse model Sapap3^−/−^	Transcriptional alteration	Soto et al. (2023)
ASD and FXS	Human iPSC	Modulation of neurons	Allen et al. (2022)
OCS in preHD	Mouse model R6/2	Electrophysiological changes	Yu et al. (2018)
GTS	Human	Transcriptional changes	de Leeuw et al. (2015)
Epigenetics			
OCD	Human	Chromatin remodeling	Lin et al. (2022)
	Mouse model Mir137^+/−^	Post-transcriptional changes	Cheng et al. (2018)
ASD and FXS	Mouse model Shank3^−/−^	Histone modification	Mahgoub et al. (2014) and Rapanelli et al. (2022)
GTS	Human	Hypermethylation	Müller-Vahl et al. (2017) and Zilhão et al. (2015)

### 3.1 Obsessive-compulsive disorder

OCD is a neuropsychiatric disorder marked by unwanted obsessions and/or compulsions, impacting approximately 1–3% of the general population (Fawcett et al., 2020). Alongside factors affecting both prenatal and postnatal life such as complications during pregnancy, labor (Geller et al., 2008), or experiencing trauma and stressful life events during childhood (Wang et al., 2023), its development is associated with genes interacting with dopaminergic and glutamatergic systems (Pauls et al., 2014). Dysfunctions in subcortical regions have been observed both in mouse models of the pathology and OCD patients (Piras et al., 2015; Zike et al., 2017). In animal studies, focal lesions to the striatum generates OCD-like behaviors (Graybiel and Rauch, 2000). Other evidence has been found in the Slitrk5 (SLIT and NTRK-like protein-5, a neuronal transmembrane protein involved in central nervous system development) knockout (KO) mouse model of OCD, where authors report a reduction in striatal volume (Shmelkov et al., 2010), evidence that recapitulates human findings (Rosenberg et al., 1997). From a molecular point of view, transcriptome analysis comparing OCD patients to healthy controls identified the caudate and the NAc as the brain areas with the highest numbers of differentially expressed genes, predominantly belonging to pathways involved in synaptic neurotransmission and glutamatergic synapses (Piantadosi et al., 2021). However, besides striatal alterations, other subcortical areas display anatomical and functional abnormalities. Both left and right thalamic areas have been shown to be significantly increased in volume in OCD patients (Rotge et al., 2009). Concerning animal evidence, in rats which underwent the signal attenuation model, a behavioral approach used to induce compulsive behavior, high frequency deep brain stimulation of rodent equivalents of the human GPi, GPe and STN, induce a reduction in compulsions (Klavir et al., 2009; Djodari-Irani et al., 2011). Moreover, inactivating these same areas in the same animal model with muscimol, a gamma-aminobutyric acid receptor (GABAr) agonist, also led to decreased compulsive behavior. Given the involvement of the striatum in repetitive behaviors, authors wondered whether an unbalance between habitual and goal-directed behavioral control could partially underlie and explain OCD symptoms. It has been found that individuals with OCD experience impairments in cognitive flexibility and impaired balance between habitual and goal-directed behavior resulting in difficulty in regulating both repetitive behaviors (compulsions) and unwanted thoughts (obsessions; Gillan et al., 2011). Devaluation tasks and other decision-making tests conducted on OCD patients highlighted a shift toward habitual control of behavior (Gillan et al., 2011, 2014; Voon et al., 2015; Barzilay et al., 2022). In rodents, researchers showed that rats that relied on a habitual behavioral strategy in a Y-maze task were the ones that developed schedule-induced polydipsia (Gregory et al., 2015), a ritualistic compulsion characterized by excessive fluid intake (Zike et al., 2017). However, other studies point to different dysfunctions besides reliance on habitual strategy. Seiler and colleagues explored in a mouse model of compulsions that shows punishment-resistant reward seeking, whether this phenotype could arise from habit formation or from strengthening of action-outcome associations in the DMS (Seiler et al., 2022). According to their findings, it is dopaminergic signaling in the DMS that precipitates compulsions. Indeed, optogenetic activation of dopaminergic terminals in the DMS accelerates compulsions onset while optogenetic inactivation delays them. The same experiment was performed in the DLS leading to no changes in the behavioral phenotype. Interestingly, manipulation of DMS did not affect inflexible behavior involving a contingency reversal test. These results suggest that dopamine (DA), by enhancing the predicted value of reward seeking actions, promotes them in face of punishment. Moreover, they also highlight that habitual behaviors and punishment resistance do not necessarily arise together. Lastly, from other clinical evidence, mechanisms favoring OCD-like manifestations seem to involve deficits in choosing the correct action-chunk to perform, which is a higher level GDB deficit (Dezfouli and Balleine, 2012; Geddes et al., 2018; Morris and Cushman, 2019). Notably, obsessive-compulsive-like symptoms are also commonly observed in individuals diagnosed with ASD (van Steensel et al., 2011; Scahill et al., 2014; Hartmann and Millet, 2018) and HD (Oosterloo et al., 2019).

### 3.2 Autism spectrum disorder and Fragile X syndrome

ASD is a neurodevelopmental disorder associated with approximately 600–1,200 genes and non-genetic factors such as parental age, maternal nutrition, exposure to environmental pollutants and preterm birth (Gaugler et al., 2014; De Rubeis and Buxbaum, 2015; Long et al., 2019; Zhong et al., 2020; Crump et al., 2021). It can feature intellectual disability, repetitive movements, adverse reactions to change, and a limited range of interests pursued in an obsessional manner (Handbook of serious emotional disturbance in children and adolescents Marsh and Fristad, 2002). Research has revealed the involvement of the corticostriatal circuitry in the manifestation of these behaviors (Wilkes and Lewis, 2018) and functional connectivity magnetic resonance imaging (MRI) studies find evidence of increased striatal connectivity with limbic cortex and reduced striatal connectivity with frontoparietal and motor circuits (Abbott et al., 2018; Soghomonian, 2023). As reviewed in Thabault et al. (2022), several ASD animal models which display inflexible and repetitive behaviors are affected by alterations at the level of the basal ganglia structures. Neuroligin3 Knock-In, Shank3^−/−^ and Cntnap2^−/−^ mice ranging from adolescence to adulthood, present bigger striatum when compared to control littermates, while BTBR model of idiopathic ASD display a reduction in this brain area. Moreover, increased volume of the NAc is found in 16p11^+/−^ mice. Structural abnormalities have been described in mouse models of Fragile X Syndrome (FXS). The most prevalent inherited cause of ASD is FXS (Moskowitz et al., 2020; Reisinger et al., 2020). FXS is caused by a mutation in the Fragile X Messenger Ribonucleoprotein 1 (FMR1) gene, responsible for coding the Fragile X Messenger Ribonucleoprotein. Loss of this protein leads to synaptic connectivity alterations due to its role in mRNA transportation and local protein translation (Grossman et al., 2006), and development of repetitive or restricted behaviors (Moskowitz et al., 2020; Reisinger et al., 2020). Studies in toddlers with FXS showed structural changes such as enlarged caudate volume and abnormal connectivity measured as lower fractional anisotropy subcortical structures (including the thalamus, basal ganglia, and cerebellum) to the PFC suggesting the involvement of the striatum in the manifestation of repetitive behaviors (Razak et al., 2020; Shen et al., 2022). However, while the specific neural circuitry underlying repetitive behaviors in FXS is yet to be elucidated, the basal ganglia and related subcortical structures appear to have a significant role in the expression of these behaviors (Hollander et al., 2005; Langen et al., 2014). Concerning impairments in habitual behavior, using a devaluation paradigm as a proxy to measure behavioral inflexibility, Alvares et al. measured a slight reduction in flexible goal-directed action control in ASD patients when compared with healthy controls (Alvares et al., 2016). Moreover, a recent paper by Mercaldo and colleagues describes increased striatal-dependent behavioral inflexibility in Fmr1^y/−^ mouse model of FXS. By testing them in the Cognition Wall task, in which mice are subjected to a discrimination and a reversal learning phase, FXS mice took more time to adjust their behavioral performance in the reversal learning phase, suggesting behavioral inflexibility. These results were confirmed by the use of another paradigm, the Touchscreen operant platform (Mercaldo et al., 2023). These findings collectively underscore the significance the corticostriatal circuit in understanding the neural basis of repetitive and possible habitual behaviors in ASD.

### 3.3 Huntington’s disease

Another inherited neurological disorder characterized by progressive cognitive impairment, sudden unintended and uncontrollable movements, and obsessive-compulsive symptoms (OCS) is HD (Rosenblatt, 2007; Kalueff et al., 2016; Ghosh and Tabrizi, 2018). HD is caused by a trinucleotide repeat expansion of the Huntingtin gene (HTT), and gradually leads to neuronal degeneration. While the advanced degeneration of the basal ganglia and the cortex is clearly associated with choreatic motor impairments, obsessive and compulsive symptoms are experienced by 20–50% of HD patients (Anderson et al., 2010). These symptoms may predate or coincide with motor impairments (Molano-Eslava et al., 2008). We focus on pre-motor HD to extrapolate striatal mechanisms underlying the obsessive-compulsive symptoms. Studies on premanifest HD (preHD) patients revealed that the striatum is one of the brain areas that shows the earliest impairments. Loss of striatal gray and white matter (Tabrizi et al., 2011; McColgan et al., 2015) have been described, along with connectivity alterations. PreHD patients display reduced striatal volume (Glikmann-Johnston et al., 2021), reduction in functional connectivity between the striatum and the motor cortex, and increased connectivity between the striatum and the PFC. Indeed, alterations of the dorsolateral PFC are associated with excessive doubt and repetitive actions in OCD (Mataix-Cols et al., 2003; Abramowitz et al., 2009). In light of OCS in preHD, where cell loss is less apparent, molecular studies could unveil the mechanisms involved in early circuit dysfunction. Huntingtin plays a pivotal role in striatal neuronal survival by stimulating cortical production of brain-derived neurotrophic factor and repressing pro-apoptotic molecules such as caspase-3 and 9 (Lebouc et al., 2020). In a transgenic mouse model, HTT has been deleted in direct and indirect SPNs at around embryonic day 16. In the juvenile 2-month-old conditional KO mouse, lack of Htt in iSPNs decreases dramatically the GABAergic synapses in the GPe. This structural feature is associated with behavioral hyperactivity. Conversely, when Htt is deleted in dSPNs, authors observed an increased inhibition of the SNr and hypoactivity. These results therefore suggest that Htt is required for the maintenance of basal ganglia circuit integrity (Burrus et al., 2020). All these human and rodent evidence are not directly linked to obsessive-compulsive manifestations; however, they highlight possible synaptic, cellular and anatomical alterations leading to OCS, especially when considering that iSPNs show earlier signs of degeneration than dSPNs (Reiner et al., 1988; Deng et al., 2004; Langfelder et al., 2016).

### 3.4 Gilles de la Tourette syndrome

GTS is a childhood onset disorder diagnosed solely by the presence of intrusive, and recurrent motor and vocal tics that, having a spontaneous rather than learned aspect, can provide further insights into the neurobiology of repetitive behaviors. What drives the emergence of complex tics is still unknown, but clinical data implies a habit component to it (Leckman and Riddle, 2000). Several neuroimaging studies have shown that patients suffering from GTS display structural and functional changes in cortico-striatal pathways, which could be linked to brain development, since GTS patients display delayed functional maturation of cortico-basal ganglia in childhood (Church et al., 2009) and adulthood (Worbe et al., 2012). Using fractional anisotropy to map microstructural axonal alterations, Worbe and co-workers reported abnormally enhanced structural connectivity of the motor cortex with the striatum and the thalamus in GTS, which was strongly correlated with tic severity (Worbe et al., 2015). Moreover, they also showed an increased structural connectivity of thalamo-putaminal regions in GTS patients, which was again strongly associated with severity of tics. These findings suggest that in GTS repetitive behavior arises from dysfunctional development and processing of motor networks within the corticostriatal loop. One other useful piece of evidence shows how GTS incorporates components of repetitive behaviors comes from a recent molecular study in which authors checked for similar gene enrichments in pathology datasets of OCD and GTS and found a strong positive correlation, suggesting that many biological functions are similarly altered in the two conditions. Among the common deregulated functional biological pathways, genes related to the terms FMRP target, RBFOX target, neurodevelopmental genes, ion channel activities, voltage-gated calcium channel activity appear to be the one showing the strongest correlation, Notably, according to their findings, authors speculate that both OCD and GTS could be developmental disorders (Lin et al., 2022). At the molecular level, Lennington and co-workers, performing transcriptome studies on postmortem samples of GTS patients, have found reduced expression of D1 and D5 dopamine receptors in the striatum (Lennington et al., 2016). We can speculate that a higher direct pathway activity during childhood, as described by Baym et al. (2008), may be compensated for by the downregulation of D1 receptors in adulthood, as noted by Lennington et al. (2016). Moreover, the same study also reported downregulation of several genes related to striatal GABAergic interneurons, including *NOS1, NPY, and SST* (Lennington et al., 2016). In fact, GTS could be best understood by looking at alterations at the cellular level, more specifically in the population of GABAergic and cholinergic striatal interneurons. Indeed, a reduced number of fast spiking interneurons in the striatum might selectively lead to the disinhibition of the direct pathway over the indirect pathway (Conceição et al., 2017). Detailed evidence regarding interneuron alterations related to GTS can be found in Section 5.1.

### 3.5 Open loop circuits: a hypothesis of striatal communication?

The aforementioned pathologies share common symptoms of repetitive behaviors and are characterized by alterations in the same neural circuitry despite different phenotypes (Gillan et al., 2011; Figures 2A–D). But as the evidence for corticostriatal circuit alterations in these pathologies is extensive and mapped out some years ago, we turn to the role of the downstream nigrostriatal pathway that has been proposed for decades to regulate repetitive behaviors and is only recently being re-investigated. Many reviews restate that the striatal regions must communicate in a ventral to dorsal flow, possibly through SNr and SNc connections with SPNs, and that disruptions in this circuit might lead to imbalanced behavioral control or repetitive behaviors (Yin and Knowlton, 2006; Lerner, 2020; Lüscher et al., 2020). This is based on the work of Haber et al. (2000). In their original theory, they propose that disinhibition of DA signals in the DLS could promote a shift from GDB to inflexible behavior. Called the ‘ascending spiral circuit’, the DLS and DMS are suggested to communicate in a polysynaptic circuit: SPNs in the DMS inhibit SNr GABAergic neurons which project to the SNc, disinhibiting DA firing from the SNc to the DLS. The reverse of this, being DLS modulation of the DMS, was termed the ‘descending spiral circuit’. Open-loop circuits are an attractive idea that could explain cross talk between the regions, and the selection and management of behavioral strategies.

The initial evidence for the ascending spiral circuit arose in a series of retrograde and anterograde tracing studies in non-human primates in the striatum and the SN (Haber et al., 2000). Axon terminals from the medial striatum appeared to overlap with cell bodies in the SN that projected to the DLS, but DLS neurons did not overlap with SN neurons that projected back to the DMS. Therefore, more emphasis was placed on the ascending spiral circuit; though the anatomical work is limited by a lack of functional evidence confirming synaptic connections between these neurons. The existence of open loops has been recently reported by Ambrosi and Lerner (2022), but their functional purpose is still unknown. They combined optogenetic stimulation, transsynaptic tracing and electrophysiology to reveal functional synapses in both ascending and descending spiral circuits. In the ascending spiral circuit, 50% of DLS-projecting DA neurons were monosynaptically inhibited by stimulation of GABAergic neurons in the SNr that are targets of DMS SPNs. In the descending spiral circuit, 45% of DMS-projecting DA neurons were monosynaptically inhibited by SNr GABAergic neurons that are targets of DLS SPNs. Suppression of their tonic firing was not observed in either circuit, meaning that disinhibition of DA in the striatum is unlikely despite the connectivity. This dissociation raises the question of the purpose of open-loop circuits. Ambrosi and Lerner (2022) suggest in open-loop circuits that the synapses could be located on distal dendrites of striatal neurons to explain the lack of firing modulation. Inputs to distal dendrites are still essential for the integration of signals in the neuron, but other excitatory inputs, perhaps from the cortex, may also be required to reach threshold activation. Additionally, SNc projections to the VMS were not investigated, though included in Haber and colleagues’ model (Haber et al., 2000). Incorporating a deeper level of detail in the cellular and molecular striatal mechanisms appears necessary to identify other root causes responsible for behavioral regulation.

## 4 Cellular and molecular mechanisms of repetitive behavior

### 4.1 Direct and indirect pathway dysregulation in repetitive behavior

The classic view in the field sees the direct pathway as the facilitator of action by disinhibiting the thalamus, while the indirect pathway suppresses action by inhibiting it (Albin et al., 1995; Calabresi et al., 2014). In this conceptual framework, Varin et al. (2023), tracked behavioral patterns such as fast locomotion, turning, grooming and sniffing in freely moving mice while recording activity of dSPNs and iSPNs. They reported that natural activation and silencing of direct and indirect pathway neurons, observed by one photon Ca^2+^ imaging, underlies behavior encoding. The findings support the notion that the coordinated activity of both dSPNs and iSPNs is necessary for the formation of neuronal representations of spontaneous behavior (Varin et al., 2023). Other studies also found with *in-vivo* fiber optic recording that the co-activation of iSPNs and dSPNs occurs during movement and ceases during immobility (Cui et al., 2013), and that their simultaneous activation facilitates movement while optogenetic inhibition disrupts it (Tecuapetla et al., 2014, 2016). Together, this evidence suggests that iSPNs are not necessarily more active during immobility and may have a role other than in action suppression. At the very least, it indicates that balanced patterns of activity between the pathways are necessary for movement control. Imbalance in the direct and indirect pathways has been proposed to underlie several movement disorders and implicated in the development of repetitive behaviors (Lovinger, 2017; Wang et al., 2017, also reviewed in Brandenburg et al., 2020; Soghomonian, 2023).

To better understand the complex neural mechanisms underlying repetitive behaviors, many circuit-based strategies target these behaviors in rodent models of disease. These include tics, self-grooming or complex restricted behaviors such as resistance to change, cognitive rigidity to routines, and obsessions (Lopez et al., 2005), and are observed in models of ASD, OCD, HD and GTS and involve striatal pathways (Figures 2A–D; Whitehouse and Lewis, 2015; Kim et al., 2016; Hartmann and Millet, 2018; Oosterloo et al., 2019).

In a Shank3B-deficient model of ASD characterized by repetitive self-grooming causing self-injury and social interaction deficits, authors suggested dysfunction in the striatopallidal pathway as the underlying cause (Wang et al., 2017). They observed distinct changes between the striatal pathways in this autism model; iSPNs show impairments in pre-and postsynaptic synaptic functions, synaptic plasticity, and spine density compared to dSPNs. To restore iSPNs activity, authors selectively enhance the indirect pathway through the expression of a Gq-coupled human M3 muscarinic receptor. Following this manipulation, repetitive self-grooming behavior activity was attenuated, suggesting that disruption of the indirect striatal pathway possibly plays a causative role in repetitive behavior in this specific context (Wang et al., 2017).

In another mouse model in which Shank3 exon 11 is deleted (Shank3 ^Δ11/Δ11^), authors report striatal transcriptional alterations in both dSPNs and iSPNs with downregulated genes mainly expressed by dSPNs and upregulated genes expressed by iSPNs (Ferhat et al., 2023). Running a functional analysis, among the protein functions primarily affected by transcriptional alterations there are synaptic transmission and G-protein activity. Authors speculate that these might underlie the aforementioned impairments in long-term depression in iSPNs but not in dSPNs (Wang et al., 2017). Similarly, in a different ASD model induced by valproic acid (VPA), which affects global gene expression and leads to ASD-like syndrome, differential alterations in striatal direct and indirect pathways were observed (Di et al., 2022). Authors report that a decreased basal iSPN activity played a role in the repetitive behaviors and that their optogenetic activation leads to decreased self-grooming behavior in this ASD model. These effects were not observed in dSPNs.

Another aspect to be considered are macro-and micro-structural alterations in the striatum and connected brain areas. A recent work exploited human data from patients presenting ASD-like phenotypes, such as stereotypies, that identified *de novo* variants in the intersectin1 (ITSN1) gene, to generate a mouse model in which intersectin genes (ITSN1 and ITSN2) are knocked down (Vollweiter et al., 2023). This mouse model displays behavioral anomalies measured as increased repetitive jumping or stereotypic running-like behavior at the wall of the cage, as well as decreased striatal, pallidal and thalamic volumes measured by structural MRI. Moreover, striatal SPNs show a lower degree of complexity of their dendritic arborization and decreased spine density, suggesting these morphological alterations could negatively impact on the size of the aforementioned brain regions. Morphological changes are accompanied by impaired basal synaptic transmission at cortico-striatal synapses, increased frequency of spontaneous excitatory synaptic currents, decreased AMPA/NMDA ratio and reduced long-term depression (LTD). At a molecular level the doubleKO shows reduced protein levels of NR2A/B NMDA receptors, PSD95 and actin. Authors do not investigate cell-type specific impairments; however, they speculate that the decrease of striatal volume and the presence of stereotypies might be due to the differential loss of either specifically dSPNs or iSPNs in this brain area (Vollweiter et al., 2023).

Further evidence implicates altered structural plasticity of striatal neurons in behavioral inflexibility phenotype of FXS mouse model. Defects at the spine level and behavioral abnormalities are rescued by increasing actin polymerization by RAC1 activity (Mercaldo et al., 2023) specifically in the striatum. Furthermore, cell-type specific alterations have been taken into account in the Fmr1 KO mouse, a model of FXS characterized by RRB, marble-burying, nestlet shredding tests and self-grooming behavior. Longo and colleagues report dysregulated *de novo* protein synthesis specifically in dSPNs, due to an increased *de novo* cap-dependent translation (Longo et al., 2023). Moreover, they find increased spine density in dSPNs of the DLS in the Fmr1KO mouse while no changes are detected in iSPNs. These structural changes are accompanied by an alteration in the frequency of miniature excitatory postsynaptic currents in dSPNs and aberrant LTD at corticostriatal pathway. Authors report a reduction of Rgs4 (Regulator of G protein signaling 4) and speculate that the alterations they observe occur via the interaction between Rgs4 and muscarinic receptor 4 (MR4). MR4 are known to facilitate dSPNs LTD and reverse aberrant plasticity associated with levodopa-induced dyskinesia. Indeed, positive allosteric modulators of this receptor are used to treat an animal model of this condition (Shen et al., 2015). Moreover, these compounds have been observed to normalize enhanced protein synthesis and mGluR-mediated LTD in the hippocampus in a Fragile X syndrome mouse model (Thomson et al., 2017). Indeed, behavior abnormalities including RRB, excessive grooming and altered LTD, are rescued in this mouse model by administration of VU0152100, a positive allosteric modulator of MR4 (Longo et al., 2023).

The acetylcholine neurotransmitter system has also been implicated in the behavioral alterations present in the SAPAP3 KO mouse model of OCD. Malgady et al. (2023) show that in DLS neurons belonging both to the direct and indirect pathway, intrinsic somatic excitability is increased, as well as firing frequencies in response to depolarizing current injections, rheobase currents are lower, and input resistances are higher. However, only dSPNs display enhanced dendritic excitability, possibly impairing the delicate balance between the direct and indirect pathway and ultimately leading to maladaptive behavioral phenotypes through aberrant muscarinic signaling (Malgady et al., 2023). The hypothesis of an imbalance between dSPNs and iSPNs could also be applied to the study performed by Petroccione and co-workers (Petroccione et al., 2023). Authors investigated the role of the neuronal glutamate transporter EAAC1, encoded by *slc1a1*, an OCD candidate gene (Zike et al., 2017). Even if EAAC1 is similarly expressed in striatal dSPNs and iSPNs, authors report an effect of its manipulation only in dSPNs. Lack of EAAC1 leads to increased dendritic branching, spine number and size. This in turn increases excitation onto dSPNs also through decreased homosynaptic lateral inhibition between dSPNs. Excitation/inhibition imbalance has been addressed also through other approaches. One example is the D1CT mouse model, which displays repetitive behaviors, in which hyperactivity of the direct basal ganglia pathway is mimicked by expression of cholera toxin in dSPNs (McGrath et al., 1999). Chronic optogenetic activation of the orbitofrontal cortex (OFC) to VMS connection induces an increase in grooming (Ahmari et al., 2013) while enhancing feed-forward inhibition of fast-spiking striatal interneurons driven by lateral OFC optogenetic activation on hyperactive SPNs rescues compulsive behaviors (Burguière et al., 2013). Another recent paper found that optogenetic inhibition of both dSPN-specific SNc projections to the VMS and SNc projections directly to the lateral OFC prevented excessive grooming (Xue et al., 2022).

The conundrum relative to whether it is an increased excitation or a reduced inhibition playing a part in the appearance of OCD symptoms was addressed by Rădulescu et al. (2017) combining dynamic system theories, therefore considering the interaction of multiple processes at different levels, and computational approaches (Rădulescu et al., 2017). The authors investigated how global and local changes in excitatory/inhibitory balance of SPNs regulate the activity of the cortico-striato-thalamo-cortical (CSTC) pathway. Specifically, the authors discriminate among more global changes in oscillations and synchronous activity in distinct domains of the CSTC pathway, which have been described to contribute to habitual control of behavior (Stern et al., 1997; White, 1997; Berke et al., 2004), and local changes driving hyperactivation of specific areas therefore leading to OCD symptoms (Saxena and Rauch, 2000; Ting and Feng, 2011). According to currently available models and previously mentioned experimental and clinical evidence, a reduction in the activity of dSPNs together with an increase in iSPNs activation leads to motor cortex inhibition. Conversely, if dSPNs are more active and iSPNs less engaged, then movement is promoted via activation of the motor cortex, possibly contributing to motor symptoms of OCD. Their findings suggest that the increase in dSPN activity, as well as the decrease in iSPN activity, can be due to changes both in excitation and inhibition. However, authors show how the oscillatory activity is much more affected by changes in GABAergic inhibition than changes in the overall activity of SPNs. Therefore, the authors reasoned that alterations in the relative inhibition among direct and indirect SPNs represent a valuable mechanism that might contribute to disrupted habit formation and movement execution in OCD.

Cell-type specific vulnerability has been described especially for HD. Disinhibition of iSPNs is believed to underlie chorea and impaired behavioral control, hallmark symptoms of HD (Bates et al., 2015; Sebastianutto et al., 2017; Callahan et al., 2022). Involvement of iSPNs in HD pathology is conserved across humans and mice. Although loss of iSPNs contributes to late-stage symptoms of chorea, there are changes in behavior that occur even before the degradation of iSPNs (Blumenstock and Dudanova, 2020). For example, the acquisition of new motor skills like repeating sequences is impaired early in HD (Heindel et al., 1988; Willingham et al., 1996). Obsessive-compulsive-like behaviors have also been characterized in otherwise preHD patients (Beglinger et al., 2008). These impairments indicate early striatal dysregulation, but the exact mechanisms underlying this symptomology are unknown. As in the ASD model (Wang et al., 2017), functional and morphological changes to iSPNs occur in early, pre-motor HD models R6/2 and zQ175-KI, such as increased iSPN excitability associated with a reduction in dendrites (Sebastianutto et al., 2017). Despite this evidence, dSPNs appear to be affected in the preHD model YAC128, as well as in the early-stage HD (1.5 months; Galvan et al., 2012). Authors describe an increased AMPA/NMDA ratio while no changes are detected in iSPNs. They also find a higher percentage of calcium-permeable AMPA receptors at the synapse that might in part explain the increased glutamatergic synaptic strength onto dSPNs (Chambon et al., 2023). However, more work is required to link cell-type specific SPN alterations to symptoms of repetitive, or OCS in HD.

There is a paucity of information concerning the contribution of dSPNs and iSPNs in the development of tics in the context of GTS. Nevertheless, the therapeutic profile of antipsychotics, which have a fast-acting effect on tic reduction (Shapiro and Shapiro, 1968), suggests a blockade of postsynaptic D2 receptors, which would suppress tics by increasing the activity of SPNs in the indirect pathway. Moreover, in a study that evaluated cognitive performance and functional brain imaging in GTS children vs. typical children, tic severity in GTS children was positively correlated with the activation of brain nuclei associated with the direct pathway, namely striatum, GPi, thalamus and motor cortex (Baym et al., 2008). At the molecular level, Lennington and co-workers, in one of the few transcriptome studies with postmortem samples of GTS patients, reported reduced expression of D1 and D5 dopamine receptors in the striatum (Lennington et al., 2016). The apparent discrepancies between the two above-mentioned studies may reflect the varying patterns of alterations in striatal dopamine observed in patients with GTS. Specifically, higher direct pathway activity during childhood, as described by Baym et al. (2008), may be compensated for by the downregulation of D1 receptors in adulthood, as noted by Lennington et al. (2016). But, perhaps at the cellular level, GTS could be best understood by looking at alterations in the population of GABAergic and cholinergic striatal interneurons, since postmortem studies have revealed a substantial reduction in the number of fast spiking interneurons in GTS during childhood, that is maintained during adulthood (Kataoka et al., 2010). A reduction in GABAergic interneurons was also found when basal ganglia organoids were generated using induced pluripotent stem-cells from GTS patients (Brady et al., 2022). Indeed, reduced number of fast spiking interneurons in the striatum might selectively lead to the disinhibition of the direct pathway over the indirect pathway (Conceição et al., 2017).

### 4.2 Striosomes vs. matrix compartments: another domain supporting repetitive behavior

Adding another layer of complexity, the striatum is composed of striosomes and matrix. Striosomes are defined as wide-spread neurochemically specialized compartments surrounded by the matrix creating a labyrinth-like organization throughout the striatum (Graybiel and Hickey, 1982). dSPNs and iSPNs are equally present in both striosomes and matrix. Interestingly, they are contacted by specific input structures with anteromedial striosomes receiving afferents preferentially from limbic structures while the matrix from sensory-motor circuits (Graybiel and Matsushima, 2023). These specific anatomic connections might divide the two compartments into different behavioral aspects with matrix, which account for 80% of the striatal volume (Crittenden and Graybiel, 2011), more related to sensory-motor functions and the striosomes more related to motivation (Saka and Graybiel, 2003; Saka et al., 2004). When considering cortical afferents, OFC, anterior cingulate cortex, and insular cortex project preferentially to striosomes, while somatosensory, motor, and association cortices mainly contact the matrix (Ragsdale and Graybiel, 1990; Eblen and Graybiel, 1995; Flaherty and Graybiel, 1995; Kincaid and Wilson, 1996; Lévesque and Parent, 1998). While the contribution of striosome and matrix domains in relation to OCD remain elusive, what is known is that compulsions and repetitive behaviors are triggered when striosomal activity is increased or matrix activity is decreased in rats and monkeys (Crittenden and Graybiel, 2011). Since hyperactivation of OFC to VMS leads to repetitive behaviors, these findings could be partially explained by anatomical connections (Ahmari et al., 2013). Concerning ASD, reports in mouse models and human postmortem brain tissue show that striatal compartments exhibit pathological alterations (Kuo and Liu, 2017, 2020). Moreover, a recent study reported a correlation between the level of self-grooming and increased striatal expression of several genes, including glutamic acid decarboxylase 2 (*Gad2*; Ferhat et al., 2023), encoding GAD65, which present preferentially in axonal terminals for synthesis of GABA for vesicle release (Esclapez et al., 1994; Tian et al., 1999). Notably, Shank3^Δ11/Δ11^ and mice with a complete lack of all SHANK3 isoforms displayed an enlargement of the compartment formed by striosomes which displayed over-expressed GAD65. Authors suggest that signaling imbalance between the striosome and matrix compartments of the striatum in SHANK3-deficient mice underlie these repetitive behaviors (Ferhat et al., 2023). These data go in line with the previous study reporting that aberrant development of striosomal and matrix compartments of striatum leads to dysfunction in corticostriatal connectivity, leading to repetitive behaviors in mouse model of ASD (Kuo and Liu, 2017).

In a recent publication, Matsushima et al. (2023) analyzed the different striatal axes (direct vs. indirect and striosome vs. matrix) in grade 1 HD patients (mild motor symptoms and psychiatric and cognitive changes) and HD mice models, to assess susceptibility to neurodegeneration and transcriptomic disturbances that might explain the different symptomatology in HD over time. Transcriptomic analysis of human post-mortem tissue found a strong depletion of striosomal iSPNs and dSPNs followed by matrix iSPNs. Even if authors were able to identify cellular subtypes, they could not perform analysis of differentially expressed genes due to the limited number of cells and tissue available. Concerning animal models, the authors reported that in zQ175 and R6/2 HD models the principal alterations occur in the striosome-matrix axis, leading to the loss of SPNs’ identities. These changes were found to be more prominent than those among dSPNs and iSPNs with neurons of the indirect pathway more impaired as previously mentioned. Among iSPNs, the most affected are a recently identified cluster of “D1/D2 hybrid” cells (He et al., 2021) referred to as “outlier-D2,” followed by striosomal iSPNs (Matsushima et al., 2023).

Overall, the conflicting evidence concerning when and how these pathways are activated in physiological and pathological conditions remain unclear. Many different aspects play a role in the onset of repetitive behaviors: the proportion and strength of activation of different SPNs, the timing of their activation, the reciprocal interaction between dSPNs and iSPNs the functional and anatomical area they are embedded into, and the possible interaction they have at a circuit level. These aspects might be differentially altered in the above mentioned physiological and pathological frameworks (Figures 2A–D). Nevertheless, the shared behavioral phenotype (i.e., proneness to engage a habitual control of behavior, RRB) unmasks how an unbalance in the coordinated activity of SPNs is consistent in altered control of behavior.

## 5 Microcircuit determinants of repetitive behavior

### 5.1 Parvalbumin positive and cholinergic interneurons

The activity of interneurons in the striatum and cortex has been shown to mediate habitual and repetitive behaviors. GABAergic, fast-spiking parvalbumin positive interneurons (PV+) and tonically active cholinergic interneurons (CIN) constitute the major classes of interneurons in the striatum (Burke et al., 2017). PV+ are diverse regarding the medial-lateral axis, where PV+ cells in DMS display increased intrinsic excitability and a lower rheobase when compared to PV+ cells in the DLS (Monteiro et al., 2018). Having a more excitable PV+ interneuron in the DMS could provide an efficient control of SPNs during an initial trial-and-error learning phase, while less excitable PV+ cells in DLS would favor the preservation of a learned behavior strategy that is effective. This hypothesis is supported by the fact that PV+ interneurons from DMS, but not from DLS (Monteiro et al., 2018), receive direct glutamatergic inputs from the cingulate cortex, a region associated with top-down control of behavior. Though it should be noted that chemogenetic inhibition of PV+ cells in the DLS prevents habitual responding (O’Hare et al., 2017), and that these DLS interneurons could play a central role in the acquisition of chunked behavioral sequences (Martiros et al., 2018). CIN are the main source of acetylcholine in the striatum (Zhou et al., 2002; Abudukeyoumu et al., 2019; Prager and Plotkin, 2019). They are implicated in the modulation of striatal circuitry through their intrinsic properties as well as the excitatory, inhibitory, and neuromodulatory inputs that they receive (Pisani et al., 2007; Goldberg et al., 2012; Gonzales and Smith, 2015). They are also good candidates to modulate habitual behavior (Cachope and Cheer, 2014; Cai and Ford, 2018). Some of the evidence, however, is somewhat contradictory. Selective lesioning of CIN in the DLS following extensive training in a cued maze task was shown to increase habitual responding (Ashkenazi et al., 2021). In another study, ablation of CIN in the DLS did not increase or change habitual lever-pressing behavior (Aoki et al., 2018). In a review by Apicella (2017), it is noted that many studies report CIN alterations in the DMS, rather than DLS, and subsequent behavioral inflexibility. This combined evidence suggests that both classes of interneurons could be involved in habitual behaviors, but the literature is still developing. As some repetitive behaviors involve a learned habit component, specific evidence of interneurons in pathologies of repetitive behaviors could expand the understanding of their role in behavioral control.

In OCD and particularly in Sapap3^−/−^ mice, optogenetic activation of striatal terminals from the lateral OFC rescues grooming behaviors possibly by enhancing GABAergic function of PV+ interneurons (Burguière et al., 2013). Kaleuff and colleagues note that as the Sapap3^−/−^ mouse seems to affect corticostriatal rather than thalamostriatal circuitry (Wan et al., 2014), the rescue of the grooming phenotype emphasizes the corticostriatal microcircuit as the driver of repetitive behaviors in the CSTC (Kalueff et al., 2016). Conversely, ablation of striatal CIN results in increased functional connectivity between the cortex and the motor, but not associative regions of striatum, and spontaneous repetitive behaviors and compulsive social behaviors (Martos et al., 2017). This could indicate that reductions in CIN and PV+ activity exacerbates OCS.

A positron-emission tomography study revealed widespread reduction in GABA_A_ receptor binding in the striatum, thalamus, and insular cortex of GTS patients (Lerner et al., 2012). Postmortem analysis showed a significant reduction in GABAergic interneurons in the striatum of GTS patients (Kalanithi et al., 2005). A reduction in GABAergic interneurons was also found when basal ganglia organoids were generated using induced pluripotent stem-cells from GTS patients (Brady et al., 2022). These findings suggest that reduced GABAergic inhibition in the striatum could be one mechanism underlying tics. In fact, local infusion of GABA_A_ antagonists in the motor areas of the striatum can produce tics and repetitive stereotypical behaviors both in mice (Pogorelov et al., 2015) and non-human primates (Worbe et al., 2013). Treatment with GABA-receptor agonists could be a possible treatment mechanism, but interestingly, in ASD, human clinical trials and mouse studies of Fmr1 KO have failed to rescue repetitive behaviors with GABA-receptor agonists (Schaefer et al., 2021). Like GTS, reduced numbers of PV+ cells have been found in the cortex (Peñagarikano et al., 2011). and decreased PV protein expression in the striatum (Lauber et al., 2018) of Cntnap2 KO mice (ASD mouse model). In humans, reductions in PV+ Chandelier cells in the cortex was associated with RRB and motor stereotypies (Dufour et al., 2023). In a different approach, a recent study found that PV+ cells in the DMS of Cntnap2, Shank3 and VPA mice are surrounded by perineuronal nets (extracellular matrix structures), and their degradation restored repetitive behaviors to normal levels (Briones et al., 2022). This mechanism could possibly occur by increasing PV+ inhibition onto SPNs. The consistent lack of efficacy of GABA-receptor agonists is therefore a noteworthy finding.

Loss of CIN in humans with GTS has also been shown in the associative and sensorimotor regions of the striatum but not the limbic regions (Kataoka et al., 2010). It is known that tics are worsened by episodes of acute stress, and ablation of CIN in the DLS followed by acute stress revealed tic like episodes in mice (Xu et al., 2015). Similarly, in ASD it has been reported that levels of the acetylcholine precursor have been decreased (Friedman et al., 2006; reviewed in Koevoet et al., 2022), while characteristic ASD-like behaviors were ameliorated after administration of cholinergic agents (Rapanelli et al., 2023). Interestingly, co-depletion of both CIN and PV+ putative neurons in the dorsal striatum resulted in stereotypy in male but not female mice, which points toward the predominance of ASD and GTS in males (Rapanelli et al., 2017).

In HD, loss of GABA interneurons is associated with mid to late-stage motor symptoms, and CIN were shown to be largely spared (Reiner et al., 2013). In another study in R6/2 mice, neurons in the parafascicular nucleus of the thalamus, which provide inputs to CIN, are involved in motor symptoms when degenerated (Crevier-Sorbo et al., 2020). Mouse models of HD show morphological and changes in synaptic plasticity of CIN as well as reduced acetylcholine release (Vetter et al., 2003; Picconi et al., 2006; Deng and Reiner, 2016). How CIN could be involved specifically in OCS in HD has yet to be explored. Overall, this evidence seems to implicate a role for both cortical and striatal PV+ interneurons, and striatal CIN in repetitive behaviors of these pathologies, but more work should be conducted in the specific molecular and physiological mechanisms of these interneurons to resolve questions arising from conflicting ablation studies, and a lack of treatment efficacy for GABA-receptor agonists in some of these conditions. Alterations of PV^+^ and CIN interneurons associated with the different pathological conditions considered in this review are summarized in Figures 2A–D.

### 5.2 Astrocytes

A surge of interest has also emerged regarding the modulation of neuron-driven behavior by astrocytes. Astrocytes are glial cells found abundantly throughout the striatum. They influence behaviors by altering neuronal activity and synaptic plasticity (Lyon and Allen, 2022). Neuron-astrocyte communication primarily occurs through G-protein coupled receptors (GPCRs) on astrocytes (Kofuji and Araque, 2021). GPCR activation leads to downstream signaling cascades, which includes the phospholipase C (PLC)/inositol 1,4,5-triphosphate (IP3) pathway, resulting in calcium release from the endoplasmic reticulum (Agulhon et al., 2008; Sobolczyk and Boczek, 2022). The increase of calcium influences the release of gliotransmitters like ATP, D-serine, and glutamate, which then exert their effects on neurons (Parpura et al., 1994; Halassa and Haydon, 2010; Sild and Van Horn, 2013; Pirttimaki et al., 2017). Studies are emerging that indicate a role for astrocytes in diseases characterized by repetitive behavior. For instance, they may be involved in HD-related circuit dysfunction (reviewed in detail elsewhere: Khakh et al., 2017; Blumenstock and Dudanova, 2020). Repetitive grooming behaviors expressed in mouse models of early-onset HD have been likened in similarity to human-phenotypes of OCS (Steele et al., 2007; Ahmari, 2016). In a mouse model of early-onset HD disease, reduced astrocytic calcium signaling resulted in decreased GABAergic tonic inhibition of SPNs in the DLS and increased self-grooming behaviors compared to controls; behavior was restored to control levels following administration of a GABA transporter 3 (GAT-3) inhibitor (Yu et al., 2018). While the authors do not distinguish between dSPNs and iSPNs, they suggest that the net effect of increased activity of GAT-3 resulting in decreased ambient GABA could be the mechanism by which astrocytes affect repetitive behavior. Other studies report that astrocytes affect neuronal activity through upregulated clearance of GABA and glutamate from the synaptic cleft (Murphy-Royal et al., 2017; Mahmoud et al., 2019; Semyanov and Verkhratsky, 2021; Paniccia et al., 2022). Astrocytic-regulated glutamate clearance through the expression of excitatory amino acid transporters (EAAT1, EAAT2; Zhou and Danbolt, 2013; Divito and Underhill, 2014; Aida et al., 2015; Sharma et al., 2019) was recently reported to shape the transition from flexible to inflexible behavior (Boender et al., 2021). Also in GTS, genetic studies have proposed altered neuron-astrocyte communication. de Leeuw et al. (2015), by performing gene-set analysis on a previously published GWAS study (Scharf et al., 2013), reported the association of GTS with 33 genes involved in astrocytic glycolysis and glutamate metabolism, which are key for the support of synaptic function.

Altered activity of astrocytes has also been linked to OCD independent from HD, as well as ASD (Figures 2A,B). SAPAP3, a protein part of the scaffolding complex at the post-synaptic density expressed in the striatum and associated with repetitive behaviors, was recently also detected at high levels in striatal astrocytes (Soto et al., 2023). Soto and colleagues utilized a SAPAP3 KO mouse model (Welch et al., 2007), typically used to study OCD-like phenotypes, that display grooming behaviors to the point of the appearance of skin lesions. They found that when SAPAP3 was rescued in the striatal astrocytes with an AAV it ameliorated grooming behaviors to a similar degree as striatal neurons. Astrocytes cultured from induced pluripotent stem cells of ASD individuals transplanted into the brains of healthy mice produced repetitive behaviors, operationalized by the marble burying test (Allen et al., 2022). Aside from the evidence suggesting the impact of striatal astrocytes in repetitive behavior, new reports propose that astrocytes from PFC are as well implicated in the development of repetitive behaviors (Petrelli et al., 2023). Collectively, these data suggest that astrocytes may play an equally important role as neurons in mediating repetitive behaviors and could be targets of therapeutics.

## 6 Beyond genetics: epigenetic players in pathological repetitive behavior

The term epigenetics comprises a variety of molecular mechanisms that enable cells to adapt to environmental changes. These mechanisms range from DNA and RNA methylation, histone modifications and various species of non-coding RNA such as miRNAs and long non-coding RNAs. In the past decade, few studies investigated the role epigenetics in the regulation of physiological and maladaptive habitual behaviors (Langen et al., 2011; Shiflett and Balleine, 2011; Malvaez et al., 2018). While extensive evidence has been gathered about the development of maladaptive habit formation upon drug abuse and its effect on synaptic plasticity (Ostlund and Balleine, 2008; Corbit et al., 2014; Nestler, 2014; Nestler and Lüscher, 2019), less information is available concerning repetitive behaviors (Farmer and Lewis, 2023). Epigenetic modifications of chromatin offer a mechanistic link between the genome and environmental experiences given their ability to alter the chromatin landscape, promptly adapting the molecular and cellular substrates to the requests of everyday life. For this reason, they appear particularly appealing when investigating repetitive behavioral control in physiological and pathological contexts especially in those disorders in which heritability cannot fully explain the etiology of the disease (Figure 2).

Class I HDACs (histone deacetylase) have been found implicated in disorders characterized by stereotypic and repetitive movements such as Rett Syndrome, a neurodevelopmental pathology that shares symptoms with ASD. HDAC1/2 are histone deacetylases that act as transcriptional corepressors inducing a closed chromatin state and have been extensively studied for their role in negatively regulating memory processes, especially HDAC2, representing a possible target in neurodegenerative diseases (Mahgoub and Monteggia, 2014). These players are known to interact with MeCP2, the predominant cause of Rett Syndrome (Jones et al., 1998; Nan et al., 1998; Haberland et al., 2009). In their study Mahgoub and Monteggia illustrate a connection between MeCP2, HDAC1/2 and Sapap3. They show that the conditional deletion in the postnatal mouse forebrain of both *Hdac1* and *Hdac2* leads to excessive grooming through the dysregulation of *Sapap3* in striatum via MeCp2. They conclude that lack of both *Hdac1* and *Hdac2* or the absence of their partner *MeCP2* in the forebrain are responsible for repetitive behaviors possibly deregulating *Sapap3* expression (Mahgoub and Monteggia, 2014; Lamothe et al., 2023). A recent paper took advantage of a Shank3-deficient model of ASD characterized by repetitive behaviors to test whether manipulation of histone 3 lysine 4 demethylation (H3K4me2) histone modification could ameliorate behavioral phenotypes. Indeed, H3K4me2 is a histone mark linked to permissive gene transcription that has been found decreased in the PFC of both ASD patients and mutant mice with the deficiency of Shank3 or Cul3. Authors show that by pharmacologically inhibiting Lysine-specific demethylase 1 (LSD1), the enzyme that removes methyl groups from H3K4, they were able to rescue both behavioral and cellular alterations. After treatment, Shank3-deficient mice display a significant reduction in repetitive behaviors and normalization of abnormal NMDA receptor function in PFC and AMPA receptor-mediated currents in the striatum (Rapanelli et al., 2022). Besides histone deacetylases, other epigenetic enzymes have been implicated in the etiopathology of disorders implicating repetitive behaviors. A recent study used Whole Genome Sequencing (WGS) to examine DNA samples from a cohort of 53 families with 54 OCD probands and their unaffected parents and found that among the genes preferentially affected by *De Novo* Mutations there are the chromatin modifiers enzymes SETD5 (SET-containing-domain 5), KDM3B (lysine demethylase 3B), and ASXL3 (transcriptional regulator 3). Authors investigated the concomitance of mutations in these genes encoding for epigenetic modifiers with deregulation of genes belonging to glutamate, serotonin, and dopamine neurotransmitter systems such as tyrosine hydroxylase, monoamine oxidase A and serotonin 1D receptor among the others. They found that co-expression patterns between these neurotransmitter systems and chromatin modifiers are significantly altered in the PFC in patients with OCD raising the possibility that alterations in the epigenetic landscape might be upstream of neurotransmitter system impairments ultimately leading to the onset of an obsessive phenotype (Lin et al., 2022).

GTS is a disease largely influenced by inheritable polygenetic factors (de Leeuw et al., 2015; Mataix-Cols et al., 2015), although environmental factors, like heavy smoking during pregnancy, low weight at birth, premature birth and older paternal age have also been associated with disease development and severity of symptoms in some studies (Mathews et al., 2006; Hoekstra et al., 2013; Browne et al., 2016; Brander et al., 2018). Understanding how environmental factors impact genetics in GTS can lead to a better understanding of the etiology of the disease. In this context, using bisulfite sequencing to assess peripheral DNA methylation in adult GTS patients, Müller Vahl and co-workers found increased DNA methylation of the D2 receptor gene, with a strong association between hypermethylation and tic severity, corroborating to the “dopamine hypothesis” in GTS (Müller-Vahl et al., 2017). However, this study did not explore the impact of this epigenetic modification on gene expression, nor has investigated what is the impact of methylation of specific cytosine residues on D2 receptor expression. Another study, performing whole blood genome wide epigenetic analysis in a Dutch population, fail to show any significant genome-wide association between CpG island methylation and tic disorders (Zilhão et al., 2015). Nevertheless, employing a less conservative *p*-value, they found that top CpG island hits were enriched in brain-specific and developmental processes ontologies. Interestingly, 2 of the top CpGs were located near the GABBR1 gene, which codes a GABA receptor subunit. These studies provide further mechanistic insights for the understanding of neurotransmitter dysfunction in GTS.

Considering this evidence, besides behaviorally-induced or genetic causes of repetitive behavior, it has to be kept in mind the power of epigenetic mechanisms in the pathogenesis of or in the definition of the progression of the behavioral phenotype. As mentioned, few studies highlight the role of chromatin modifying enzymes such as histone deacetylases. However, also miRNAs have been described as associated with RRB in ASD (Hicks et al., 2020). miRNAs could have the ability to influence habit formation as they have been found able to modulate addiction-relevant plasticity genes like *Arc* (Quinn et al., 2015, 2018). One study reported that the partial deletion of the psychiatric risk gene *mir137* in mice induces repetitive and impairs social behaviors associated with ASD, via upregulation of the phosphodiesterase 10a (PDE10A; Cheng et al., 2018). This enzyme is highly enriched in the striatum, and papaverine, a potent PDE10A inhibitor, has been shown to increase striatal output and therefore has been proposed as an antipsychotic drug (Siuciak et al., 2006). Taken together, these studies suggest that striatal inhibition of PDE10A could underlie the normalizing effect of papaverine over the repetitive behavior in mir137 conditional KO mice. Thus, the investigation of epigenetic contribution to the physiology and etiopathology of repetitive behaviors is a critical node to better understand the connection between genetic and behavioral alterations thus still requiring efforts to be thoroughly addressed.

## 7 Discussion

The striatum is at the center of repetitive pathological behaviors. Its cells rely on neurotransmitters GABA to function in circuits with the cortex, interneurons, and output nuclei of the basal ganglia. Neuromodulators also affect these behaviors (though reviewed in detail elsewhere), as well as astrocytes and epigenetic modifications which we identify as therapeutic targets in future research.

CSTC circuitry is altered in pathological repetitive behavior through changes in signaling and synaptic activity. The exact causes of these alterations have been a focus of this research area for decades. We discussed recent advances in identifying cellular and molecular striatal mechanisms that could address this gap. We found several pieces of evidence of punctual molecular and synaptic alterations affecting preferentially dSPNs, iSPNs or both, all leading to the onset of repetitive behaviors. It is, however, difficult to reconcile all these experimental findings on a lower scale (cellular dysfunction). What appears to be clear about the role of the direct and indirect pathways is that they require balanced activation, as an imbalance is associated with pathological manifestations. The threshold and determinants of pathway imbalance could differ between pathologies, between striatal domains (regions and compartments), and between individuals. Moving to a higher scale, manipulating many different aspects of the circuitry, at the level of the cortex, striatum or output nuclei appears to produce similar phenotypes, indicating the delicacy of the system and the importance of the entire circuit. All this suggests the need to consider multiple layers of investigations in single studies, together with the implementation of computational modeling, where single biologically-relevant parameters can be adjusted returning their net effect on the whole network.

On the other hand, peculiar dysfunctions represent optimal therapeutic targets. For example, a new role for a subclass of iSPNs, known as outlier-D2, was identified as most affected in HD as they show the highest degree of cell-type-specific dysregulation of gene expression (Matsushima et al., 2023). Identifying specific subclasses of SPNs with a specific molecular profile might make them ideal candidates to target for therapeutic purposes. This line of thinking extends to distinct striosomal populations which have been linked to repetitive behaviors in HD, ASD and OCD. With altered glutamate and GABA metabolism, as well as altered calcium signaling, leading to repetitive behaviors, long-neglected astrocytes appear to also be good therapeutic candidates. Epigenetic changes are based on one’s exposure to the environment, but they can occur even earlier in life like when a child is developing *in utero* and is affected by the mother’s environment. For conditions like ASD, in which some forms are long ago concluded to develop from genetics (FXS, Rett syndrome), but many others are hardly attributable to clear genomic mutations, transgenerational environmental influences through epigenetics might be a worthy route to be explored. Epigenetic targets are also emerging as possible treatment avenues. Finally, it is true that both habitual behaviors, and repetitive behaviors more broadly, can involve pathology or not, such as when they become counterproductive or debilitating. The overlap in these differently defined behaviors can be found within the research on pathologies OCD, ASD, HD and proposed a working model of GTS, which can be comorbid. We suggest a unified approach to studying repetitive behaviors and maladaptive habitual behavior to form a complete picture of the mechanisms underlying both. Setting a common framework could help to advance improvements in therapeutic interventions and treatment regimes. Overall, this subject demands an equal understanding of multiple layers of complexity to achieve a comprehensive view of repetitive behavior and the rules of cooperation among its players.

## Author contributions

CB: Conceptualization, Writing – original draft, Writing – review & editing. AL: Conceptualization, Writing – original draft, Writing – review & editing. AZ: Writing – original draft, Writing – review & editing. GG: Writing – original draft, Visualization, Writing – review & editing. RT: Conceptualization, Funding acquisition, Supervision, Writing – original draft, Writing – review & editing.

## Glossary

**Table:** 

ASD	Autism spectrum disorder
CIN	Cholinergic interneurons
CSTC	Cortico-striato-thalamo-cortical
DA	Dopamine
D1	Dopamine receptor 1
D2	Dopamine receptor 2
DLS	Dorsolateral striatum
DMS	Dorsomedial striatum
dSPN	Direct spiny projection neuron
FXS	Fragile X syndrome
GABA	Gamma-aminobutyric acid
GDB	Goal-directed behavior
GTS	Gilles de le Tourette syndrome
HD	Huntington’s disease
HDAC	Histone deacetylase
iSPN	Indirect spiny projection neuron
ITSN	Intersectin
KO	Knock out
LTD	Long-term depression
MRI	Magnetic resonance imaging
NAc	Nucleus accumbens
OCD	Obsessive-compulsive disorder
OCS	Obsessive-compulsive symptoms
OFC	Orbitofrontal cortex
PFC	Prefrontal cortex
preHD	Premanifest Huntington’s disease
PV+	Parvalbumin positive
RRB	Restricted and repetitive behaviors
SNc	Substantia nigra pars compacta
SNr	Substantia nigra pars reticulata
SPNs	Spiny projection neurons
VMS	Ventral medial striatum
VPA	valproic acid
VTA	Ventral tegmental area
VS	Ventral striatum
